# Effects of vibration training vs. conventional resistance training among community-dwelling older people with sarcopenia: three-arm randomized controlled trial protocol

**DOI:** 10.3389/fnagi.2022.905460

**Published:** 2022-09-01

**Authors:** Linqian Lu, Xiangfeng He, Lin Ma, Yu Liu, Nan Chen

**Affiliations:** ^1^Department of Rehabilitation, Xinhua Hospital Chongming Branch, Shanghai, China; ^2^Key Laboratory of Exercise and Health Sciences of Ministry of Education, Shanghai University of Sport, Shanghai, China; ^3^Department of Rehabilitation, Xinhua Hospital Affiliated to Shanghai Jiaotong University School of Medicine, Shanghai, China

**Keywords:** sarcopenia, vibration training, resistance training, muscle mass, muscle strength, physical performance, blood biomarkers

## Abstract

**Introduction:**

Sarcopenia is a chronic and progressive disease, which is accompanied by the decline in muscle mass, muscle strength, and physical performance with aging, and it can lead to falls, fracture, and premature death. The prevention and treatment of sarcopenia mainly include exercise therapy and nutritional supplement. Exercise therapy is one of the most potential interventions to prevent and/or delay the progression of sarcopenia. Resistance training (RT), one of the most commonly used exercise types, is widely used in the treatment of sarcopenia, while vibration training (VT) is a prospective strategy for improving sarcopenia in older people. The aim of our study is to compare the effect of VT and RT in older people with sarcopenia on muscle mass, muscle strength, physical performance, blood biomarkers, and quality of life.

**Methods and analysis:**

Our study is a 12-week, three-arm randomized controlled trial with assessor-blinded. The diagnosis criteria for subject recruitment adopt the guidelines for the Asian Working Group for Sarcopenia. A total of 54 subjects who met the criteria were randomized into one of the following three groups: VT group, RT group, and control group. The VT group and RT group received a 12-week whole-body vibration training and a resistance training program three times every week, respectively. The primary outcome is lower limb muscle strength, and the secondary outcomes include muscle mass, upper limb muscle strength, physical performance, blood biomarkers, and quality of life. We then performed assessments three times, at baseline (0 week), after intervention (12 weeks), and follow-up (24 weeks). The adverse events were also be reported. All outcome measurements were performed by the same researchers. Data were saved in the unified database, and the collected data of all subjects were analyzed by intention-to-treat analysis.

**Ethics and dissemination:**

This study was reviewed and approved by the Ethical Committee of Xinhua Hospital Chongming Branch. The findings of the study were authorized in peer-reviewed journals with online access; meanwhile, it will be presented at domestic or international academic congresses.

**Clinical trial registration:**

Chinese Clinical Trial Registry (ChiCTR2100051178), registered on 15 September 2021.

## Introduction

Sarcopenia is a progressive disease associated with aging, characterized by low skeletal muscle mass, decreased muscle strength, and/or poor physical performance (Chen et al., 2020). In China, the prevalence of sarcopenia in older people aged 60 years and above in the suburb-dwelling population was 9.3%, with 6.4% in men and 11.5% in women according to the guideline for the Asian Working Group for Sarcopenia (AWGS) (Han et al., 2016). Several adverse outcomes, such as accidental falls (Zhang et al., 2020), fractures of upper and lower limbs (Zhang et al., 2018), physical disability (Janssen et al., 2002), cognitive impairment (Peng et al., 2020), poor quality of life (Tsekoura et al., 2017), or even death (Nascimento et al., 2019), are associated with the progression of sarcopenia. The changes of older people with sarcopenia are not only reflected in clinical outcomes (Chen et al., 2020) and physiological outcomes, such as age-related inflammation (Tournadre et al., 2019), metabolic abnormalities (Hunter et al., 2019), and endocrine changes (McKee et al., 2017). The pathophysiological mechanisms of sarcopenia are complexly associated with the following blood biomarkers: inflammatory factors {i.e., hypersensitive C-reactive protein [hs-CRP] (Shokri-Mashhadi et al., 2021), tumor necrosis factor-α cytokines [TNF-α] (Bian et al., 2017), and interleukin-6 [IL-6] (Rong et al., 2018)}, hormones {i.e., insulin growth factor-1 [IGF-1] (Ascenzi et al., 2019), growth hormone [GH] (Bian et al., 2020), testosterone (Colla et al., 2015), and 25(OH)D (Remelli et al., 2019; Abiri and Vafa, 2020)}, growth factors {i.e., myostatin [MSTN] (White and LeBrasseur, 2014) and follistatin [FST] (Echeverria et al., 2021)}, and muscular injury biomarkers such as creatine kinase (CK) (Kurita et al., 2021). The adverse outcomes of sarcopenia inflict a huge economic burden to patients and their families (Bruyère et al., 2019). Therefore, some measures should be taken to prevent and/or delay the development of the sarcopenia.

Until now, there is no particularly effective pharmacological treatment for sarcopenia (Cruz-Jentoft and Sayer, 2019), and many studies have indicated that non-pharmacological treatment is the most potential treatment, especially exercise training, and results in significant improvements in muscle function and physical fitness in older people with sarcopenia (Lo et al., 2020). Currently, one of the most commonly used exercise trainings for older people with sarcopenia is resistance training (RT) (Beckwée et al., 2019). Vibration training (VT), a prospective strategy for improving sarcopenia in older people (Wu et al., 2020), is a training method that uses the vibration platform with different vibration frequencies and amplitudes in different positions (Nordlund and Thorstensson, 2010). In recent years, the application of VT has been more widely used to delay the loss of muscle functions in older people with sarcopenia (Wei and Ng, 2018; Camacho-Cardenosa et al., 2019; Zhu et al., 2019). A meta-analysis demonstrated that VT can change the declining trend of muscle mass, muscle strength, and physical performance (Wu et al., 2020); meanwhile, several studies have confirmed that VT has positive effects on sarcopenia (Wei et al., 2016, 2017; Chang et al., 2018; Wei and Ng, 2018). Thus, VT may be a potential alternative training method to improve sarcopenia compared with conventional resistance training (CRT), which is generally a high-intensity RT. Although a large number of studies have confirmed the benefits of VT on muscle function, there is still a lack of comparison of the effects between VT and CRT in older people with sarcopenia. For VT to replace CRT, it is necessary to compare the effects of the two training methods on sarcopenia in older people.

RT is the most effective method to improve muscle function and physical performance in older people. The American College of Sports Medicine (ACSM) (Nelson et al., 2007) and the World Health Organization (World Health Organization, 2018) recommend 2–3 times of RT (60–80% of one repetition maximum [1RM]) per week. The intensity of RT recommended by the ACSM is ≥ 70% 1RM to achieve significant muscle hypertrophy among older people (American College of Sports Medicine, 2009). Similarly, it is demonstrated that high-intensity RT (60–70% 1RM) can produce significant effects on muscle conditions and physical performance in older people (Chen et al., 2017; Guizelini et al., 2018; Sahin et al., 2018) compared to low-intensity RT (40% 1RM) (Lixandrão et al., 2017; Sahin et al., 2018). However, RT needs high-technology requirements for older people (Wernbom et al., 2008; Thiebaud et al., 2014) and it is easy to produce muscle fatigue (Jacko et al., 2018). These may reduce the compliance of older people, especially for frail older people, and are difficult to generally conduct in the clinical setting.

Thus, the aims of our study are to (1) evaluate the effects of 12-week VT and RT on muscle mass, muscle strength, physical performance, blood biomarkers, and quality of life in older people with sarcopenia; (2) compare the effects of VT and RT on sarcopenia; and (3) explore the mechanisms of VT and RT on the improvement of sarcopenia.

## Methods and analysis

### Study design

**Figure 1:**
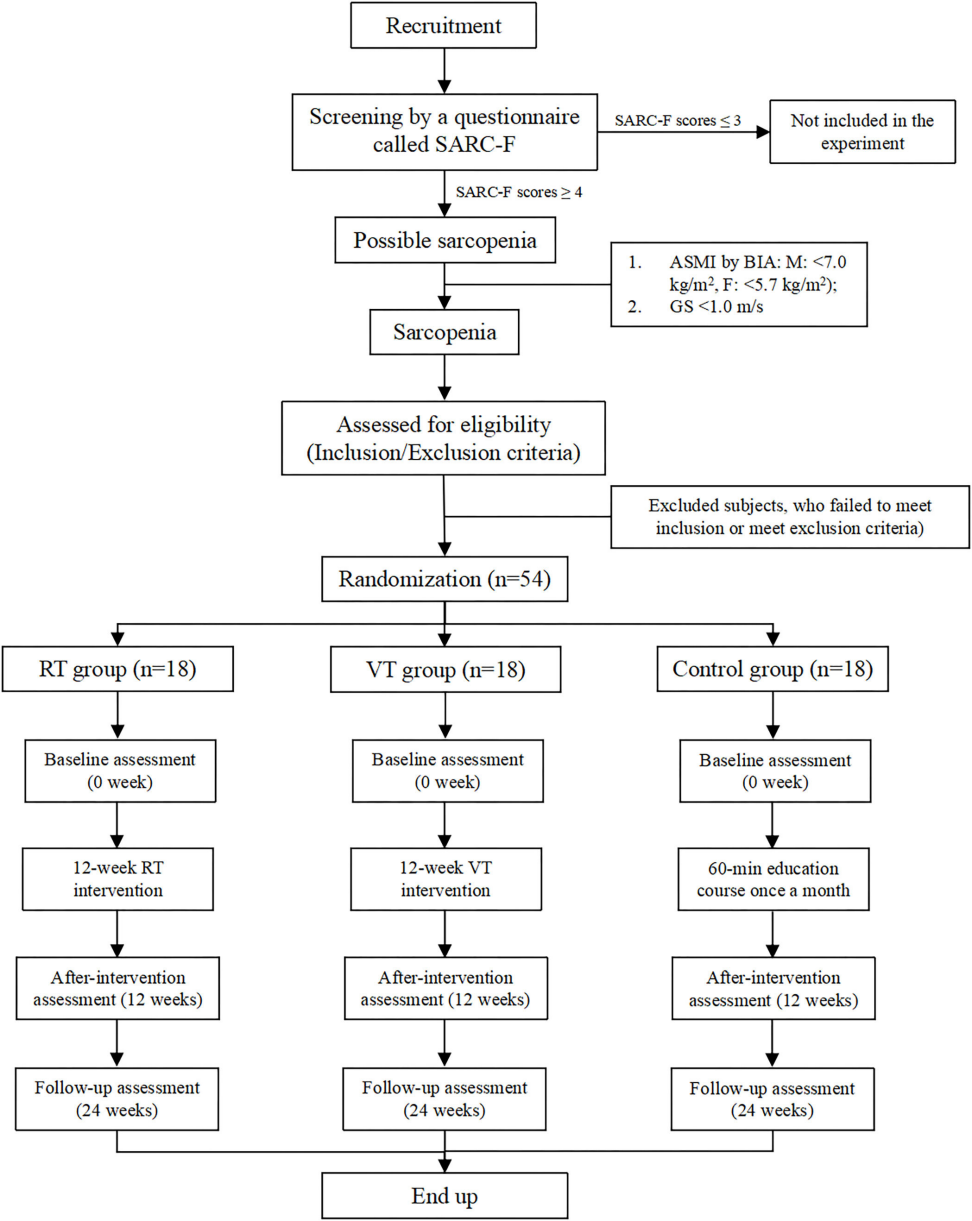
Experimental flowchart showing the patient recruitment, intervention, and assessment process.

This study is a three-arm, assessor-blinded, randomized control trial. The experimental flowchart is given in Figure 1. The schedule of enrollment, interventions, and assessments for our study is shown in Table 1.

**Table 1:** Schedule of enrollment, interventions, and assessments.

**Study period**
**Timepoint**		**Before week 0**	**Week 0**	**Week 1–12**	**Week 12**	**Week 24**
Screening	Eligibility screening	X				
Allocation	Informed consent	X				
Intervention	VT			X		
	RT			X		
	Education (No intervention)			X		
Assessment	Muscle mass		X		X	X
	Muscle strength		X		X	X
	Physical performance		X		X	X
	Blood biomarkers		X		X	X
	Quality of life		X		X	X
Others	Subjects' compliance		X	X	X	X
	Adverse events		X	X	X	X
	Dropout reasons			X		

VT, vibration training; RT, resistance training.

### Study setting

We selected subjects for our study from four communities in Chongming district of Shanghai, China, who met the inclusion and exclusion criteria. This study was conducted in May 2022 at Xinhua Hospital Chongming Branch. A total of 54 older people met the eligibility criteria and were enrolled in the study. The subjects were stratified by sex and then randomized into one of three groups (18 in each group): VT group, RT group, and control group. The assessments were performed three times: at baseline (0 week), after intervention (12 weeks), and follow-up (24 weeks).

### Inclusion criteria

1. People aged between 65 and 80 years;
2. Older people diagnosed with sarcopenia according to the criteria for AWGS: appendicular skeletal muscle mass index (ASMI) < 7.0 kg/m^2^ for male (M) and < 5.7 kg/m^2^ for female (F) using a bioelectrical impedance analyzer (BIA); handgrip strength (HGS), M: < 28 kg, F: <18 kg; and/or gait speed (GS) < 1.0 m/s in the 6-m walk (6-MW) test;
3. The participants without serious diseases, such as uncontrolled hypertension, hyperlipidemia, hyperglycemia, and diabetes;
4. The participants without untreated diseases, such as gallstones or kidney stones, and infectious diseases;
5. The participants with no regular exercise habit for more than 3 months.

### Exclusion criteria

1. People with absolute contraindication for exercising, such as deep venous thrombosis and/or blood clotting disorders;
2. People with serious cognitive impairments that have impact on the assessments and communication with researchers, such as dementia and mental illness;
3. Individuals suffering from serious diseases that affect the assessments and interventions, such as musculoskeletal diseases (e.g., fracture, dislocation, osteoporosis, and rheumatoid arthritis) and disability (e.g., loss of hands, feet, or limbs);
4. Individuals with serious spinal diseases or a surgical history, such as vertebral fracture, inflammatory joint disease, ankylosing spondylitis, spondylolisthesis, rheumatism, cauda equina syndrome, and tumor;
5. Individuals who have taken the following drugs regularly in past 3 months: antiplatelet agents (e.g., aspirin, Aggrenox, cilostazol, eptifibatide, ticlopidine, and tirofiban), anticoagulant drugs, and analgesic drugs;
6. Patients with other diseases whose participation in this study is not recommended by doctors;
7. Patients who did not sign the informed consent before the beginning of study.

### Termination criteria

1. The participants having serious adverse events or complications during the intervention period and cannot continue to participate in the study;
2. The participants who proposed that they were unwilling to continue to participate in the study;
3. Lost contact with subjects during the experiment and/or follow-up.

### Recruitment

The subjects were recruited using three approaches: (1) the annual community physical examination in four communities in Chongming district; (2) educational lectures and talks about sarcopenia at community centers; (3) home visits in cooperation with workers in the community, and the physical examination of older people who do not participate in community activities regularly for a long time.

### Screening

The subjects were screened by a screening questionnaire called SARC-F, which assesses five aspects in older people: strength, assistance in walking, rising from a chair, climbing stairs, and falls (Malmstrom and Morley, 2013; Malmstrom et al., 2016). The SARC-F questionnaire has a score range (0 [best] to 10 [worst]) for the initial diagnosis of sarcopenia, and older people with scores ≥ 4 points were considered as having a risk of sarcopenia (Chen et al., 2020). Then, the older people with a score ≥ 4 points underwent a further screening in accordance with the AWGS standard: (1) the assessment of HGS using a hand dynamometer (Jamar Plus+ Digital Hand Dynamometer; IL, USA), M: < 28 kg, F: < 18 kg; or (2) and five-time chair stand (5-CS) test, the time of the 5-CS test ≥ 12 s. If the results of the measurements meet the aforementioned standards, older people were considered “possible sarcopenia”. A formal assessment of sarcopenia was performed using the ASMI with a portable BIA system (Inbody, S10, Korean), M: < 7.0 kg/m^2^, F: < 5.7 kg/m^2^, and the assessment of the 6-MW test, the result of GS < 1.0 m/s.

### Randomization and blinding

The subjects were informed of the purpose of the study and the safety risks that may happen in the study, and if they were willing to participate in the study, informed consent was required to sign before the subjects were screened. A stratified randomization method by sex was performed on the subjects meeting the eligibility criteria for a balanced distribution so that each group will have the same number of males and females. The stratified randomization sampling was performed by a researcher, who did not participate in our experiment, by a computer program (Research Randomizer Form www.randomizer.org). The subjects were then randomized into three groups: VT group, RT group, or control group at a 1:1:1 ratio. The randomized number and group assignments were sent to a researcher, who also did not participate in our study, as an electronic file with encryption to store the experimental information. The therapists for different intervention groups conducted the intervention according to the group assignments. Only the therapists will not be blinded to the group assignments, and other research staff (including the assessors, data managers, and data statisticians) will be blinded to the group assignments.

### Interventions

#### Group protocols

A pre-training for the VT group and RT group were performed three times to familiarize them with the corresponding training courses. Formal training was conducted after pre-training. In two intervention groups (VT ang RT groups), 5-min warm-up and cool-down training were conducted before and after intervention. All training sessions were carried out by physical therapists. During the whole training period, the researchers recorded the participants' attendance and compliance. If a subject could not participate in the training on time due to some reasons, training was performed at other times in that week. All subjects were asked not to perform additional physical activity and/or change their daily lifestyle during the experimental period.

#### Vibration training program

The VT group received a 30-min whole-body vibration training (WBVT) three times a week for 12 weeks. For the WBVT, a vibration platform generating vertical vibrations (Siehe Typenschild, Wellengang excellence, Germany) was used. Each training lasted about 30 min, which included a 5-min warm-up, 20-min vibration training, and 5-min cool-down. The vibration frequency was progressively increased to 20–25 Hz and the peak-to-peak amplitude of 4 mm. During the WBVT, the subjects was asked to stand barefoot on the vibration platform with knees flexion at 30°, and a vibration rope, if needed, were held by the subjects with both hands to maintain balance. And the movement in WBVT should maintain 1 min, and repeat 10 groups with 1-min resting between groups in each training day.

#### Resistance training program

The RT group received a 12-week resistance exercise program for three times every week. Each exercise session included three parts: (1) a 5-min warm-up (mainly stretching) of the neck, shoulders, lower back, hips, knees, and ankles; (2) a resistance exercise training, which consisted of three sets/10 reps of each exercise session, and the subjects had a 1-min rest between sets. The TheraBand elastic band is used for the resistance exercise training. Before beginning the intervention, each subject should complete the 1RM test to evaluate muscle strength and determine the intensity of resistance exercise training. According to the results of the 1RM test, the intensity of resistance exercise training was specified according to the individual's 1RM. The intensity was 60% 1RM in the first 4 weeks, 65% 1RM in the second 4 weeks, and 70% 1RM in the third 4 weeks. Resistance exercise movements include upper limb exercises (elbow flexion, shoulder extension, and abduction), lower limb exercises (hip abduction, knee flexion, and extension), and trunk exercises (chest press); and (3) a 5-min cool-down was performed after the aforementioned exercise training, which is similar to the warm-up exercise.

#### Control group with education courses

The subjects in the control group received a 60-min education course two times a month at the community center. The education course was taught by the invited experts, and the topics of courses were aging, health, and sarcopenia, including the definition of sarcopenia, cause of disease, pathology, clinical performance, and adverse consequences for older people.

#### Nutritional intake control

During a 12-week intervention period and a 12-week follow-up period, all subjects were required to maintain their dietary habits. The study staff and community workers supervised and recorded the nutritional intakes, and the record content on the specific notebook includes the diet of three meals a day for protein, fat, etc. The frequency of dietary recording should be maintained at two times a week on weekdays and once on weekends.

### Outcome measurements

The outcome was measured at baseline (0 week), after invention (12 weeks), and follow-up (24 weeks).

#### Primary outcome

Lower limb muscle strength was assessed by an estimated 1RM of knee extension strength (KES) (Abdalla et al., 2020). The 1RM test was the most popular method used for measuring muscular strength, but the standard 1RM test was too difficult for frail older people with sarcopenia and can even cause the injury to subjects (Rontu et al., 2010); therefore, our study chose the estimated 1RM test, which is more suitable to assess muscle strength for frail older people. The calculation is as follows: Estimated 1RM (kg) = submaximal weight (kg)/(1.0278–0.0278×maximal number of repetitions) (Brzycki, 1993). The subjects should sit on the extensor chair and the weight plates of extensor chair are in kilograms (Brzycki, 1993). In the beginning, the weight was set at M: 64% of the body mass and F: 45% of the body mass (Kuramoto and Payne, 1995; Abdalla et al., 2020). If a subject can perform knee extension 10 times under the initial weight, the weight was increased, and the repetitions of the maximal weight was recorded. Conversely, if the subject repeated 10 times or less, the weight was considered the submaximal weight. The maximal number of repetitions performed and the submaximal weight were used to calculate the estimated 1RM.

#### Secondary outcomes

##### Anthropometric measurements

Body height (BH) and body weight (BW) were measured by researchers. BH measurement requires the subjects to stand barefoot on the floor with the standard posture for measuring height. When measuring BW, the subjects were required to wear minimal clothes and stand barefoot on the digital weight scale. In addition, the calf circumference (CC) or other basic physical indicators of the subjects were measured and recorded.

##### Muscle mass

Muscle mass includes skeletal muscle mass (SMM), skeletal muscle mass index (SMI), appendicular skeletal muscle mass (ASM), and ASMI. These indicators were measured using the BIA (Inbody, S10, Korean). The SMI and ASMI were calculated (SMI= SMM/height^2^, kg/m^2^; ASMI= ASM/height^2^, kg/m^2^).

##### Upper limb muscle strength

HGS as an indicator of upper limb muscle strength was assessed by a Jamar^®^ hand dynamometer (Jamar Plus+ Digital Hand Dynamometer; IL, USA). The measurement process is as follows: in a standing position, the subjects were instructed to hold a dynamometer with the upper limb abducted at 30° from the body. The maximum HGS of each hand was measured and recorded continuously for three times, and the maximum value was considered as the final HGS value.

##### Physical performance

Physical performance was assessed using the Short Physical Performance Battery (SPPB) test, 6-MW test, and the Timed Up and Go (TUG) test.

The SPPB test includes a progressive standing balance test, a walk test, and a chair sit-to-stand test (Welch et al., 2020). In the balance test, the subjects were asked to stand in a side-by-side, semitandem and full-tandem positions, respectively. The subjects must stand at each position for at least 10 s. In the walk test, the subjects were asked to take a 4-m usual walk at the preferred GS from the standing position, and the tests were repeated two times, and the fastest time was selected. In the chair sit-to-stand test, the subjects were asked to fold their arms across the chest (without using one's arms) and try to complete five-time chair sit-to-stand tests as rapidly as possible.

Using the 6-MW test to assess GS, the subjects must walk 6 m at their usual speed. The test was repeated two times with the 2- to 3-min resting time, and the average value was calculated as the final GS.

The procedure of the TUG test is as follows: the subject should initially sit in the chair, stand up from the armchair (with the arms crossing in front of the chest), walk ahead 3 meters (at normal speed and as fast as possible), bypass the obstacle, walk back, and then sit down again (Podsiadlo and Richardson, 1991). The test was performed two times with a 2- to 3-min resting time, and the mean value was recorded as the final record.

##### Blood biomarkers

For each participant, the fasting venous blood sample after 12-h overnight fasting was collected by nurses in the morning about 08:00 for baseline (0 week), after intervention (12 weeks), and follow-up (24 weeks). The blood sample was sent to the laboratory for an automatic enzyme-linked immunosorbent assay to determine the level of blood biomarkers. The blood biomarkers include inflammatory factors (hs-CRP, TNF-α, and IL-6), hormones [IGF-1, GH, testosterone, and 25(OH)D], growth factor (MSTN and FST), and a muscular injury biomarker (CK).

### Quality of life

Quality of life in older people was assessed using the validated Chinese version of the 36-item Short-Form Health Survey (SF-36). The SF-36 consists of 36 items and eight health scales (physical functioning, role-physical, bodily pain, general health, vitality, social functioning, role-emotional, mental health, and reported health transition) assessing function and wellbeing of older people. The total score ranges from 0 to 100, and a higher score represents better quality of life.

### Sample size

In our study, we used G-Power software version 3.1 to calculated the simple size. A previous study showed that lower limb muscle strength can directly reflect the effect of exercise training on sarcopenia (Eckardt, 2016; Floreani et al., 2018). Thus, lower limb muscle strength was used as the primary outcome for the calculation of simple size. A power of 0.80 with an alpha of 0.05 was obtained in the calculation of simple size of each group, and the effect size was 0.48, which was reported in a previous meta-analysis study (Rogan et al., 2015). Our study adopted this effect size for calculating the sample size. We used above parameters in G-Power software and produced the total simple size as 45 subjects (15 subjects per group). Considering that there is a potential 20% drop rate of subjects, the total sample size should be 54 subjects (18 per group) in our study.

### Statistical analysis

The results in the study took the questionnaire result and five components of the outcome measurements into account. All results were analyzed by IBM SPSS Statistics version 24 (SPSS, Inc., USA) with a statistical significant difference as *p* < 0.05. Continuous variables were represented by mean ± standard deviation or median. The chi-square test was used for categorical variables. For baseline characteristics data of between-group comparisons, we used the one-way analysis of variance (for parametric continuous data) or Kruskal–Wallis tests (for non-parametric continuous data).

The linear mixed model was used to analyze the differences in the outcome variables between the three intervention groups, which change over time following the principle of intention-to-treat. Missing data were assumed at random and then were processed by using the linear mixed model and maximum likelihood method.

### Safety monitoring

The occurrence of adverse events during the experiment was recorded. Serious adverse events were reported to the superior unit and recorded, and then the best solution was sought for subjects by researchers. Medical treatments and appropriate medical compensation were provided to the subjects, if necessary. If the subjects thought the next intervention would cause safety problems to them, they had the rights not to continue to participate in the experiment.

### Data management and monitoring

All data in this study were recorded and managed by data management software in Microsoft Excel 365 at baseline (0 week), after intervention (12 weeks), and follow-up (24 weeks), and adverse events and solutions during the experiment were also recorded. In order to ensure the confidentiality of the study, the name of the subjects was not uploaded in the database, but an identifiable number. All researchers used the encrypted computer when recording and saving data to ensure the security of data, with back up data to avoid data loss.

The data monitoring committee (DMC), which is independent of the competing interests and sponsor, regularly monitored and tested the data to ensure the authenticity and reliability of the data. This committee comprises trial experts, clinical experts, investigators, and statisticians.

### Ethics and dissemination

#### Ethics approval

This study was reviewed and approved by the Ethical Review Committee of the Xinhua Hospital Chongming Branch in November 2020 (approval no. CMEC-2020-KT-42) and was registered in the China Clinical Trial Registry on 15 September 2021 (ChiCTR2100051178).

In the study, researchers collected demographic data (including name, age, gender, marital status, occupation, lifestyles, usual physical activity, drug use, and disease history), all questionnaire results, outcome results, and signed informed consent from the subjects. The electronic data were stored in an encrypted computer, and the paper data were be stored in the project office of Xinhua Hospital Chongming Branch.

#### Dissemination

We will authorize the publication of our study in peer-reviewed journals, and also will be performed at domestic or international academic congresses. The individual results will be reported to the subjects of this study by the researcher after publishing. Any changes or additions to the study protocol will be documented at www.chictr.org.cn.

## Discussion

This is the first study comparing the effects of VT and RT in older people with sarcopenia. In addition, we comprehensively discussed the effects of VT and RT on outcomes related to sarcopenia, including anthropometric measurements (BH, BW, and CC), muscle mass (SMM, SMI, ASM, and ASMI), muscle strength (HGS and KES), physical performance (TUG test, GS, and SPPB), blood biomarkers [hs-CRP, TNF-α, IL-6, IGF-1, GH, testosterone, 25(OH)D, MSTN, FST, and CK], and quality of life (SF-36), between two intervention groups and a control group. At the present stage, a sharply increasing proportion of older people are suffering from sarcopenia, causing many adverse outcomes [e.g., falls (Woo and Kim, 2014), fractures (Harris et al., 2017), and other secondary health problems] to older people. Increasing studies have indicated that WBVT is a potential alternative method to delay the progression of sarcopenia in older people, and RT is one of the most effective exercise training methods for sarcopenia in older people. Therefore, we aimed to find an effective, simple training method to prevent and treat sarcopenia.

Our study has the following advantages: First, our study used two training methods (VT and RT) to provide suitable treatment for older people with sarcopenia. However, CRT has the risk of increasing injury like musculoskeletal problems for older people (Keogh and Winwood, 2017). It may be that CRT is too difficult to be implemented for older people, especially for frail older people, which could lead to low participation, low adherence, and perception difficulty (Fisher et al., 2017). The practical application of the research shows that compared with CRT, VT requires less technical abilities, less space, and less time to perform training (Marín and Rhea, 2010). Compared with physically strong older people with sarcopenia, frail older people may be more appropriate for VT, rather than RT. Therefore, we used VT and RT methods for older people with sarcopenia in our study.

Second, our study compared the effects of the two training methods (VT vs. RT) in older people with sarcopenia, rather than focusing on only one training method. For example, previous studies only explored the effects of different protocols of vibration training in older people with sarcopenia (Wei et al., 2016, 2017). A systematic review and meta-analysis by Wu et al. also pointed out that current studies did not explore the efficacy of WBVT compared with other exercise training methods, and recommended more in-depth studies should be carried out in future on the comparison on WBVT with CRT (Wu et al., 2020). Therefore, our study aimed to solve this problem and provide a simple, effective alternative training method for older people with sarcopenia with difficulty to carry out the CRT.

Third, our study selected 20–25 Hz as the relatively appropriate vibration frequency. Previous studies on vibration were limited to comparing the efficacy of different vibration protocols in older people with sarcopenia. Wei et al. found that the most effective frequency of VT to improve muscle performance in older people with sarcopenia ranged between 20 and 40 Hz (Wei et al., 2016, 2017). Daniel et al. also demonstrated that the vibration frequency of about 6–26 Hz can improve GS and decrease the time of the TUG test in frail older people (Wadsworth and Lark, 2020). Stengel et al. found that VT with a frequency of 25–35 Hz produced positive effects on body composition and muscle strength in older people aged ≥ 65 years (von Stengel et al., 2012). Thus, we selected 20–25 Hz as the vibration frequency in our study. In addition, too long vibration exposure time may reduce subjectivity and compliance of older people with sarcopenia during the intervention period (Silva-Grigoletto et al., 2011). Therefore, we adopted a shorter vibration exposure time and increased the number of vibration repetitions.

Fourth, previous studies mostly focused on the acute effect of VT on sarcopenia (Giombini et al., 2013; Perchthaler et al., 2015) and less on the long-term effect of VT on sarcopenia. Thus, our study aimed to explore the long-term effects of VT and RT in older people with sarcopenia. Therefore, we set the intervention period as 3 months, and after intervention for 3 months, we performed the follow-up assessment to explore the remaining effects of VT and RT on sarcopenia in subjects.

Fifth, the subjects of our study are older people with sarcopenia but without other serious diseases. Previous studies mostly focused on young people [such as athletes (Gómez-Bruton et al., 2017; Takanashi et al., 2019; Chang et al., 2021)] and older people suffering from various diseases [such as knee osteoarthritis (Lai et al., 2019; Zhang et al., 2021), Parkinson's disease (Ribot-Ciscar et al., 2017), chronic low-back pain (Kaeding et al., 2017)], or healthy elderly (Camacho-Cardenosa et al., 2019; Jaime et al., 2019; Pérez-Gómez et al., 2020; Cheng et al., 2021). There are relatively few studies on the effects of VT on frail older people (especially for sarcopenia).

Sixth, the indicators measured in our study not only include three primary outcomes of sarcopenia (i.e., muscle mass, muscle strength, and physical performance) but also the outcomes of blood biomarkers (including inflammatory factors, hormones, growth factors, and muscular injury biomarkers) and quality of life. For the measurement of muscle strength, we measured both upper and lower limb muscle strength. In previous studies, only upper limb muscle strength (Chang et al., 2018; Miller et al., 2018) or lower limb muscle strength (Wei et al., 2016; Wei and Ng, 2018; Camacho-Cardenosa et al., 2019) was assessed. But studies have demonstrated that sarcopenia is associated with the decline of whole-body muscle, so it is not enough to only study upper/lower muscle strength (Cruz-Jentoft and Sayer, 2019). Therefore, our study evaluated both upper and lower limb muscle strength for the included subjects. For the measurement of blood biomarkers, our study explored the effects of different training methods on the physiological mechanism of the progression of sarcopenia. However, there is a lack of studies focusing on the change in the blood biomarker level in older people with sarcopenia using VT. Thus, we speculated that VT can also improve the blood biomarkers related to sarcopenia so as to prevent and/or delay sarcopenia in older people. In addition, we aimed to explore the mechanism of sarcopenia by exploring the changes of the blood biomarker level caused by VT and RT in the subjects.

Our study also has a limitation: in the VT group, we reduced each vibration exposure time and increased the number of vibration repetitions. A study found that longer time at each vibration increases muscle fatigue of the subjects and decreases subjects' compliance (Silva-Grigoletto et al., 2011). Therefore, we reduced the vibration exposure time of each vibration and increased the number of vibration repetitions. But we are not sure whether this will also lead to muscle fatigue in older people with sarcopenia. Hence, in future experiments, we will increase the monitoring of subjects' movements and make timely adjustments.

### Trial status

On 15 September 2021, this study was registered in the Chinese Clinical Trial Registry with the number ChiCTR2100051178. The recruitment of subjects began in October 2021 and ended in February 2022. The formal study is expected to start in May 2022 and end in November 2022.

## Strengths and limitations of this study

1. The findings of this study provide a convenient, safe, and effective method for older people to prevent and/or treat sarcopenia.
2. To the best of our knowledge, this study is the first to compare the effects of vibration training (VT) and resistance training (RT) on muscle mass, muscle strength, physical performance, blood biomarkers (e.g., inflammatory factors, hormones, growth factors, and muscular injury biomarkers), and quality of life in older people with sarcopenia.

## Ethics statement

The studies involving human participants were reviewed and approved by the Ethical Review Committee of the Xinhua Hospital Chongming Branch. The patients/participants provided their written informed consent to participate in this study.

## Author contributions

LL, XH, LM, and NC designed the study. XH and NC provided advice and improved the details about the vibration training and resistance training program. LL, XH, and LM drafted the manuscript. YL and NC contributed to the interpretation of the results and critical revision of the manuscript for important intellectual content, approved the final version of the manuscript, and the study guarantors. XH and LM contributed to data acquisition, analysis, and manuscript revision. All authors have read and approved the final manuscript.

## Funding

This study was supported by grants from the special health research project of Shanghai Municipal Health Commission on the Health of Ageing, Woman and Children, “Exploration on the screening and Rehabilitation intervention Model for sarcopenia among community-dwelling older people in Chongming District under the Medical Union Model” (No. 2020YJZX0137) and the project of Science and Technology Committee of Chongming District, “Effect of Exercise Intervention on Breast Cancer Patients in Chongming District” (No. CKY2021-05).

## Conflict of interest

The authors declare that the research was conducted in the absence of any commercial or financial relationships that could be construed as a potential conflict of interest.

## Publisher's note

All claims expressed in this article are solely those of the authors and do not necessarily represent those of their affiliated organizations, or those of the publisher, the editors and the reviewers. Any product that may be evaluated in this article, or claim that may be made by its manufacturer, is not guaranteed or endorsed by the publisher.

## Abbreviations

AWGS: Asian Working Group for Sarcopenia
ACSM: American College of Sports Medicine
M: male
F: female
BH: body height
BW: body weight
CC: calf circumference
BIA: bioelectrical impedance analyzer
SMM: skeletal muscle mass
SMI: skeletal muscle mass index
ASM: appendicular skeletal muscle mass
ASMI: appendicular skeletal muscle mass index
1RM: one repetition maximum
HGS: handgrip strength
KES: knee extension strength
6-MW test: 6-meter walk test
5-CS test: 5-chair stand test
GS: gait speed
SPPB: Short Physical Performance Battery
TUG test: Timed Up and Go test
hs-CRP: hypersensitive C-reactive protein
TNF-α: tumor necrosis factor-α cytokines
IL-6: interleukin-6
MSTN: myostatin
FST: follistatin
IGF-1: insulin growth factor-1
GH: growth hormone
CK: creatine kinase
SF-36: Short-Form Health Survey
RT: resistance training
CRT: conventional resistance training
VT: vibration training
WBVT: whole-body vibration training.
